# Distributed NN-Based Formation Control of Multi-Agent Systems: A Reduced-Order Appointed-Time Observer Approach

**DOI:** 10.3390/s24020589

**Published:** 2024-01-17

**Authors:** Yuting Feng, Shuai Sun, Yuezu Lv, Changhao Sun

**Affiliations:** 1 Qian Xuesen Laboratory of Space Technology, China Academy of Space Technology, Beijing 100094, China; sunchanghao@spacechina.com; 2 Beijing Institute of Control Engineering, Beijing 100190, China; sunshuai_hitsa@163.com; 3 Advanced Research Institute of Multidisciplinary Sciences, Beijing Institute of Technology, Beijing 100081, China; yzlv@bit.edu.cn

**Keywords:** reduced-order observer, appointed-time estimation, formation control, multi-agent system

## Abstract

Although the formation control of multi-agent systems has been widely investigated from various aspects, the problem is still not well resolved, especially for the case of distributed output-feedback formation controller design without input information exchange among neighboring agents. Using relative output information, this paper presents a novel distributed reduced-order estimation of the formation error at a predefined time. Based on the proposed distributed observer, a neural-network-based formation controller is then designed for multi-agent systems with connected graphs. The results are verified by both theoretical demonstration and simulation example.

## 1. Introduction

In recent years, the formation control problems of multi-agent systems have been extensively studied in various fields; see [1] and the references therein. Recently, typical perspectives on formation control include the time-varying formation control of multi-agent systems [2,3,4,5], the rigid formation of multiple robots [6,7,8], the fractional-order-based controller for multi-agent formation [9], event-based formation control [10,11] circle formation control [12], and game-based formation [13]. For the formation problem of multi-agent systems, the main task is to design an appropriate distributed controller to drive the agents form the predefined formation shape.

The distributed formation controllers are based on the feedback of either the formation error or the formation error estimation. Compared with the formation error feedback controller, the observer-based control is more practical since it does not require full state measurement of the controller. Most of the existing observer-based formation controllers rely on the exchange of the observer or input information among neighboring agents, which causes high demand for communication channels. To overcome this limitation, the unknown input observer [14] is introduced to formulate the distributed pure relative output feedback observer and generate the distributed attack-free protocols for the consensus problem of multi-agent systems [15]. To realize better performance, it is preferable to design appointed-time observers rather than asymptotical convergent ones. In view of this, the distributed appointed-time observers are introduced in [16], and the pairwise structure [17,18] is borrowed therein, which consumes double the calculation costs. Another method to construct the appointed-time observers is the time-varying transformation approach presented in [19], and the corresponding distributed appointed-time observer is proposed in [20]. Although the distributed appointed-time observer in [20] reduces the computational cost compared with those in [16], the order reduction in the distributed appointed-time observers is far from being completely resolved.

Motivated by the discussions above, this paper focuses on the reduced-order appointed-time observer and the corresponding formation controller design for nonlinear multi-agent systems. The transformation for the pairwise reduced-order appointed-time observer used in [16] is introduced, which makes it possible to further reduce the order of the designed observer. Following the observer design procedure, a novel transformation-based distributed reduced-order observer is presented, which can realize the appointed-time estimation of the formation error. Based on the proposed appointed-time observer, the distributed formation controller is then designed, where the neural network approximation is introduced with adaptive weighted gain designed to tackle the unknown nonlinearities of the agent dynamics. Theoretical analysis shows that the proposed distributed formation controller can realize the preset formation shape.

The contributions of the paper are at least twofold. Firstly, compared with existing formation results [4,5,6,7,8,10,11], this paper, for the first time, designs an output-feedback formation controller based on only relative output information, where no observer information transmission is needed during the whole process. Such a design structure has the advantages of reducing communication cost and being free from network attack. Secondly, compared with existing distributed appointed-time observers for multi-agent systems [16,20], the appointed-time observer designed in this paper is of a lower order, which decreases the computational cost.

The rest of the paper is organized as follows. Section 2 formulates the problem. Section 3 gives the main result of the paper, and Section 4 presents a simulation example to illustrate the efficiency of the proposed controller. Section 5 concludes the paper.

Notations. The symbols R and C represent the sets of all real numbers and complex numbers, respectively. The symbol $R^{n}$ is the set of *n*-dimensional real vectors. ∥M∥ represents the 2-norm of the matrix *M*. Rank(M) is the rank of matrix *M*.

## 2. Problem Formulation

Consider a distributed formation control problem of a networked system, containing *N* agents. The dynamics of the multi-agent systems are given as

(1) $$\begin{array}{cc} \; & {\dot{x}}_{i}\left(t\right)=Ax_{i}\left(t\right)+B\left(u_{i}\left(t\right)+f_{i}\left(t\right)\right),\\ \; & y_{i}\left(t\right)=Cx_{i}\left(t\right),\;i=1,\cdots ,N, \end{array}$$

where $x_{i}\left(t\right)\in R^{n}$ is the state of the *i*th agent, $y_{i}\left(t\right)\in R^{m}$ is the output of the *i*th agent, $u_{i}\left(t\right)\in R^{q}$ is the input of agent *i*, and $f_{i}\left(t\right)$ is the unknown nonlinear term satisfying the following assumption.

**Assumption** **1.** *The unknown dynamics $f_{i}\left(t\right)$ can be approximately described by*

$$f_{i}\left(t\right)=W_{i}\varphi_{i}\left(t\right)+\epsilon_{i}\left(t\right),$$

*where $W_{i}\in R^{p\times q}$ is the unknown neural network constant weight matrix; $\varphi_{i}\left(t\right)\in R^{q}$ is the known neural network activation function vector; and $\epsilon_{i}\left(t\right)$ is the residual error vector with relatively small upper bound, i.e., $∥\epsilon_{i}\left(t\right)∥\leq \Pi_{i}$. Moreover, the neural network activation functions $\varphi_{i}\left(t\right)$ are also bounded.*

The constant matrices *A*, *B*, and *C* are the dynamic matrix, the input matrix, and the output matrix, respectively.

**Assumption** **2** ([16])**.** *The matrices A,B,C satisfy*

*(i)*  rank(CB)=rank(B)=q;
*(ii)*  $\mathrm{rank}\left[\begin{array}{cc} A-sI_{n} & B\\ C & 0_{m\times q} \end{array}\right]=n+q$, ∀s∈C.

**Remark** **1.** *Assumption 2 indicates that the rank of the output matrix C is no less than that of input matrix B, and there is no transmission zero for the agent dynamics. Under Assumption 2, the distributed observer is designed in [16] without using relative input information, where the consensus error of multi-agent systems is successfully estimated at an appointed time. Note that in [16], the pairwise observer structure [17] is used, and the proposed appointed-time observer is of order 2n, which greatly increases the computational cost. To release the calculation burden, the time-varying transformation structure [19] is introduced to formulate the n-order distributed transformation-based appointed-time observer for networked systems [20].*

The communication graph among the *N* agents is described by an undirected graph G=(V,E), where V={1,2,⋯,N} is the node set and E⊂V×V is the edge set. An edge is denoted by a pair of nodes (j,i)∈E corresponding to an information link from agent *j* to agent *j*, and node *i* can have access to the relative output information $y_{i}\left(t\right)-y_{j}\left(t\right)$ via its local sensors. For the undirected graph, (j,i)∈E also means (i,j)∈E. A path from node $i_{1}$ to node $i_{k}$ is an edge sequence $\left(i_{k},i_{k-1}\right),\left(i_{k-1},i_{k-1}\right),\cdots ,\left(i_{2},i_{1}\right)$ with $(i_{l},i_{l-1})\in E,l=k,k-1,\cdots ,2$. An undirected graph is connected if for each pair of nodes i,j there exists path from node *i* to node *j*. The adjacency matrix $A={\left[a_{ij}\right]}_{N\times N}$ is defined as $a_{ii}=0$, $a_{ij}=1$ if (i,j)∈E and 0 otherwise. The Laplacian matrix $L={\left[l_{ij}\right]}_{N\times N}$ is defined as $l_{ii}=\sum_{j=1}^{N}a_{ij}$ and $l_{ij}=-a_{ij}$ when i≠j.

**Assumption** **3.** *The undirected communication graph is connected.*

Under Assumption 3, one has the following useful lemma.

**Lemma** **1.** *For the connected graph G, the Laplacian matrix L is semi-positive definite with 0 being a simple eigenvalue.*

For the multi-agent system (1), let the formation error of agent *i* be

$$\eta_{i}\left(t\right)=\sum_{j=1}^{N}a_{ij}\left[x_{i}\left(t\right)-x_{j}\left(t\right)-p_{ij}\right],$$

where $p_{ij}$ is the formation configuration between agents *i* and *j*. It is obvious that the formation is achievable if $p_{ij}=-p_{ji},\;p_{ij}+p_{jk}=p_{ik},\forall i,j,k$ and there ${\bar{u}}_{i}$ exists such that

(2) $$Ap_{ij}=B\left({\bar{u}}_{i}-{\bar{u}}_{j}\right),\;i,j=1,\cdots ,N.$$

Under condition (2), the dynamics of the formation error are given as

(3) $$\begin{array}{c} {\dot{\eta }}_{i}\left(t\right)=A\eta_{i}\left(t\right)+B\left[\sum_{j=1}^{N}a_{ij}[\left({\bar{u}}_{i}-{\bar{u}}_{j}\right)+\sum_{j=1}^{N}a_{ij}\left[\left(u_{i}\left(t\right)-u_{j}\left(t\right)\right)+f_{i}\left(t\right)-f_{j}\left(t\right)\right]\right]. \end{array}$$

The objective of this paper is to design an appropriate distributed formation controller based on output information to realize the formation of the *N* agents. Note that the formation is realized if and only if the formation error $\eta_{i}\left(t\right)$ reaches zero. To realize this objective, this paper intends to (1) design a reduced-order observer with order less than *n* to estimate the formation error $\eta_{i}\left(t\right)$ to further reduce the computational cost; (2) propose an appropriate distributed controller based on the formation error estimation.

## 3. Main Results

In this section, the reduced-order appointed-time observer is firstly designed to estimate the formation error $\eta_{i}\left(t\right)$, and the distributed neural-network-based formation controller is then proposed for each agent.

### 3.1. Reduced-Order Appointed-Time Observer Design

Since the relative input information and the nonlinearity are unknown, a transformation is needed on the formation error to eliminate the second term in the right hand of (3).

Choose matrices $B_{0}\in R^{n\times (n-q)}$ and $C_{0}\in R^{m\times (m-q)}$ such that both $\left[\begin{array}{cc} B_{0} & B \end{array}\right]$ and $\left[\begin{array}{cc} C_{0} & CB \end{array}\right]$ are of full rank. Let

$${\left[\begin{array}{cc} B_{0} & B \end{array}\right]}^{-1}=\left[\begin{array}{c} T\\ S \end{array}\right],\;{\left[\begin{array}{cc} C_{0} & CB \end{array}\right]}^{-1}=\left[\begin{array}{c} V\\ U \end{array}\right],$$

where $T\in R^{(n-q)\times n},S\in R^{q\times n}$ and $V\in R^{(m-q)\times m},U\in R^{q\times m}$. By the definition of *T*, one has $TB=0_{(n-q)\times q}$, $UCB=I_{q}$ and $VCB=0_{\left(m-q)\times (m-q\right)}$. Then, it is not difficult to derive that

(4) $$\begin{array}{cc} I_{n}= & B_{0}T+BS\\ = & B_{0}T-BUCB_{0}T+BUC\left(I_{n}-BS\right)+BS\\ = & \left(I_{n}-BUC\right)B_{0}T+BUC. \end{array}$$

Let $\zeta_{i}\left(t\right)=T\eta_{i}\left(t\right)$. Then, one can obtain that

(5) $$\begin{array}{c} \eta_{i}\left(t\right)=\left(I_{n}-BUC\right)B_{0}\zeta_{i}\left(t\right)+BUy_{\eta_{i}}\left(t\right), \end{array}$$

where

$$y_{\eta_{i}}\left(t\right)=C\eta_{i}\left(t\right)=\sum_{j=1}^{N}a_{ij}\left(y_{i}\left(t\right)-y_{j}\left(t\right)-Cp_{ij}\right)$$

is the information that can be used in the observer design. Then, the appointed-time estimation of the formation error $\eta_{i}\left(t\right)$ is achievable if the appointed-time observer for $\zeta_{i}\left(t\right)$ can be designed.

The dynamics of $\zeta_{i}\left(t\right)$ can be described as

(6) $$\begin{array}{cc} {\dot{\zeta }}_{i}\left(t\right)= & TA\eta_{i}\left(t\right)\\ = & TA\left(I_{n}-BUC\right)B_{0}\zeta_{i}\left(t\right)+TABUy_{\eta_{i}}\left(t\right). \end{array}$$

Let $y_{\zeta_{i}}\left(t\right)=VCB_{0}\zeta_{i}\left(t\right)$. Then, one has

(7) $$\begin{array}{cc} y_{\zeta_{i}}\left(t\right)= & VCB_{0}T\eta_{i}\left(t\right)\\ = & VC\left(I-BS\right)\eta_{i}\left(t\right)\\ = & Vy_{\eta_{i}}\left(t\right). \end{array}$$

That is, $y_{\zeta_{i}}\left(t\right)$ can be used in observer design.

It is known from [16] that under Assumption 2, $\left(TA\left(I_{n}-BUC\right)B_{0},VCB_{0}\right)$ is observable. Then, by borrowing the time-varying transformation structure [19,20], one can design the distributed appointed-time observer as follows:

(8) $$\begin{array}{cc} {\dot{\bar{\zeta }}}_{i}\left(t\right)= & -{\left(TA\left(I_{n}-BUC\right)B_{0}+FVCB_{0}\right)}^{T}{\bar{\zeta }}_{i}\left(t\right)\\ \; & +\left[\left({\left(VCB_{0}\right)}^{T}-G\left(t\right)F\right)V+G\left(t\right)TABU\right]y_{\eta_{i}}\left(t\right),\\ {\hat{\zeta }}_{i}\left(t\right)= & {\left(G\left(t\right)\right)}^{-1}{\bar{\zeta }}_{i}\left(t\right),\\ {\hat{\eta }}_{i}\left(t\right)= & \left(I_{n}-BUC\right)B_{0}{\hat{\zeta }}_{i}\left(t\right)+BUy_{\eta_{i}}\left(t\right), \end{array}$$

where ${\bar{\zeta }}_{i}\left(0\right)=0$, *F* is the gain matrix such that $\bar{A}=-\left(TA\left(I_{n}-BUC\right)B_{0}+FVCB_{0}\right)$ is stable, and G(t) is the time-varying transformation, which is calculated by

$$G\left(t\right)=\int_{0}^{\infty }e^{{\bar{A}}^{T}\tau }{\left(VCB_{0}\right)}^{T}VCB_{0}e^{\bar{A}\tau }d\tau$$

with G(0)=0.

Note that G(t) is the observability Gramian of the pair $\left(\bar{A},VCB_{0}\right)$, and the derivative of G(t) can be described as

$$\dot{G}\left(t\right)={\bar{A}}^{T}G\left(t\right)+G\left(t\right)\bar{A}+{\left(VCB_{0}\right)}^{T}VCB_{0}.$$

Since $\left(TA\left(I_{n}-BUC\right)B_{0},VCB_{0}\right)$ is observable, one has that G(t) is invertible. Therefore, the observer designed in the previous subsection exists under Assumption 2.

The following result shows the efficiency of the designed observer.

**Theorem** **1.** *Under Assumption 2, the distributed observer ${\hat{\eta }}_{i}\left(t\right)$ in (8) estimates the formation error $\eta_{i}\left(t\right)$ at any appointed time in the sense that ${\hat{\eta }}_{i}\left(t\right)\equiv \eta_{i}\left(t\right),\forall t>T_{0}$, with $T_{0}$ being any preset time instant.*

**Proof.**  Let $\theta_{i}\left(t\right)=G\left(t\right)\zeta_{i}\left(t\right)$. Then, the dynamics of $\theta_{i}\left(t\right)$ can be written as

(9) $$\begin{array}{cc} {\dot{\theta }}_{i}\left(t\right)= & \dot{G}\left(t\right)\zeta_{i}\left(t\right)+G\left(t\right){\dot{\zeta }}_{i}\left(t\right)\\ = & {\bar{A}}^{T}\theta_{i}\left(t\right)-\left(G\left(t\right)F-{\left(VCB_{0}\right)}^{T}\right)y_{\zeta_{i}}\left(t\right)\\ \; & \;\;\;+G\left(t\right)TABUy_{\eta_{i}}\left(t\right). \end{array}$$

Let ${\tilde{\theta }}_{i}\left(t\right)={\bar{\zeta }}_{i}\left(t\right)-\theta_{i}\left(t\right)$. Then, one has

(10) $$\begin{array}{c} {\dot{\tilde{\theta }}}_{i}\left(t\right)={\bar{A}}^{T}{\tilde{\theta }}_{i}\left(t\right). \end{array}$$

Note that ${\tilde{\theta }}_{i}\left(0\right)={\bar{\zeta }}_{i}\left(0\right)-G\left(0\right)\zeta_{i}\left(0\right)=0$, which implies ${\tilde{\theta }}_{i}\left(t\right)\equiv 0$. Therefore, one can conclude that ${\hat{\eta }}_{i}\left(t\right)\equiv \eta_{i}\left(t\right),\forall t>0$. □

**Remark** **2.** *The key point of realizing observer reduction is the introduction of the transformation T. Specifically, by introducing the variable $\zeta_{i}=T\eta_{i}$, one only has to estimate the variable $\zeta_{i}$ since $\eta_{i}$ can be reformulated by $\zeta_{i}$ and the output $y_{\eta_{i}}$; see (5). Then, the observer ${\bar{\zeta }}_{i}$ is designed to estimate the variable $G\zeta_{i}$, and thus ${\hat{\zeta }}_{i}$ can estimate the variable $\zeta_{i}$, which leads to the convergence of ${\hat{\eta }}_{i}-\eta_{i}$.*

**Remark** **3.** *The observer presented in (8) relies on the relative output information only, which overcomes the limitation of input transmission via communication topologies. Such a design structure decouples the observer design and the formation controller design, which facilitates the formation controller design. Compared with distributed n-order appointed-time observer based on the time-varying transformation structure designed in [20], the proposed appointed-time observer is of order n−q, which has the advantage of reducing the calculation cost.*

### 3.2. Distributed NN-Based Formation Controller Design

Based on the appointed-time formation error estimation, the following distributed formation controller is designed:

(11) $$\begin{array}{cc} u_{i}\left(t\right) & =-{\hat{W}}_{i}\varphi_{i}\left(t\right)+cK{\hat{\eta }}_{i}-{\bar{u}}_{i}+\frac{dK{\hat{\eta }}_{i}}{∥K{\hat{\eta }}_{i}∥+\varepsilon_{i}\left(t\right)},\\ {\dot{\hat{W}}}_{i} & =B^{T}P{\hat{\eta }}_{i}\varphi_{i}^{T}\left(t\right), \end{array}$$

where ${\hat{W}}_{i}$ is the estimation of the unknown neural network weight matrix, and *P* is a positive definite matrix with $Q=P^{-1}$ satisfying the following LMI:

(12) $$QA^{T}+AQ-2BB^{T}<0,$$

*K* is the feedback gain matrix designed as $K=-B^{T}P$, and $\varepsilon_{i}\left(t\right)$ is the damping signal satisfying

(13) $${\dot{\varepsilon }}_{i}=-k_{i}\varepsilon_{i},$$

with $k_{i}>0$ and $\varepsilon_{i}\left(0\right)>0$.

We have the following result to design the parameter *c*.

**Theorem** **2.** *Suppose that Assumptions 1–3 hold. The formation of the N agents is achieved by the distributed NN-based formation controller (11) if the parameters satisfies*

$$c>\frac{1}{\lambda_{2}\left(L\right)},\;\;d\geq \max_{i\in V}\left\{\Pi_{i}\right\},$$

*where $\lambda_{2}\left(L\right)$ denotes the smallest nonzero eigenvalue of Laplacian matrix L.*

**Proof.**  Choose $p_{k},k=1,\cdots ,N$ such that $p_{ij}=p_{i}-p_{j},\forall i,j$. Then, one has $Ap_{i}=B{\bar{u}}_{i}$. Let ${\bar{x}}_{i}=x_{i}-p_{i}$, and one has $\eta_{i}=\sum_{j=1}^{N}a_{ij}\left({\bar{x}}_{i}-{\bar{x}}_{j}\right)$. By Theorem 1, ${\hat{\eta }}_{i}\left(t\right)\equiv \eta_{i}\left(t\right)$ for $t>T_{0}$ with arbitrarily small $T_{0}$. Then, the dynamics of ${\bar{x}}_{i}$ can be written as

(14) $$\begin{array}{cc} {\dot{\bar{x}}}_{i}= & A{\bar{x}}_{i}+cBK\eta_{i}-B{\tilde{W}}_{i}\varphi_{i}\left(t\right)+B\left[\frac{d}{∥K\eta_{i}∥+\varepsilon_{i}\left(t\right)}K\eta_{i}+\epsilon \left(t\right)\right], \end{array}$$

with ${\tilde{W}}_{i}={\hat{W}}_{i}-W_{i}$. And the compact form of $\bar{x}={\left[{\bar{x}}_{1}^{T},\cdots ,{\bar{x}}_{N}^{T}\right]}^{T}$ is given by

(15) $$\begin{array}{cc} \dot{\bar{x}}= & \left[I⊗A+cL⊗BK\right]\bar{x}-\left(I⊗B\right)\tilde{W}\varphi \left(t\right)\\ \; & +\left(dI_{N}⊗B\right)m+\left(I⊗B\right)\epsilon \left(t\right), \end{array}$$

where $\tilde{W}=\mathrm{diag}\left({\tilde{W}}_{1},\cdots ,{\tilde{W}}_{N}\right)$, $\varphi \left(t\right)={\left[\varphi_{1}^{T}\left(t\right),\cdots ,\varphi_{N}^{T}\left(t\right)\right]}^{T}$, $m=[\frac{1}{∥K\eta_{1}∥+\varepsilon_{1}\left(t\right)}{\left(K\eta_{1}\right)}^{T},\cdots ,$$\frac{1}{∥K\eta_{N}∥+\varepsilon_{N}\left(t\right)}{\left(K\eta_{N}\right)}^{T}{]}^{T}$, $\epsilon \left(t\right)={\left[\epsilon_{1}^{T}\left(t\right),\cdots ,\epsilon_{N}^{T}\left(t\right)\right]}^{T}$.

Consider the Lyapunov function candidate

(16) $$\begin{array}{cc} V_{1}= & {\bar{x}}^{T}\left(L⊗P\right)\bar{x}+\sum_{i=1}^{N}\left[\mathrm{tr}\left({\tilde{W}}_{i}^{T}{\tilde{W}}_{i}\right)+\frac{2\Pi_{i}\varepsilon_{i}}{k_{i}}\right]. \end{array}$$

The time derivative of $V_{1}$ is given by

(17) $$\begin{array}{cc} {\dot{V}}_{1}= & {\bar{x}}^{T}\left[L⊗\left(PA+A^{T}P\right)-cL^{2}⊗2PBB^{T}P\right]\bar{x}\\ \; & -2\eta^{T}\left(I_{N}⊗PB\right)\tilde{W}\varphi \left(t\right)+2\eta^{T}\left(I⊗PB\right)\epsilon \left(t\right)\\ \; & +2\eta^{T}\left(dI_{N}⊗PB\right)m-\sum_{i=1}^{N}2\Pi_{i}\varepsilon_{i}\\ \; & +\sum_{i=1}^{N}2\mathrm{tr}\left({\tilde{W}}_{i}^{T}B^{T}P\eta_{i}\varphi_{i}^{T}\left(t\right)\right). \end{array}$$

Note that

$$\begin{array}{cc} \; & -2\eta^{T}\left(I_{N}⊗PB\right)\tilde{W}\varphi \left(t\right)=-\sum_{i=1}^{N}2\eta_{i}^{T}PB{\tilde{W}}_{i}\varphi_{i}\left(t\right)\\ = & -\sum_{i=1}^{N}2\varphi_{i}^{T}\left(t\right){\tilde{W}}_{i}^{T}B^{T}P\eta_{i}=-\sum_{i=1}^{N}2\mathrm{tr}\left({\tilde{W}}_{i}^{T}B^{T}P\eta_{i}\varphi_{i}^{T}\left(t\right)\right). \end{array}$$

Then, we have

(18) $$\begin{array}{cc} {\dot{V}}_{1}= & {\bar{x}}^{T}\left[L⊗\left(PA+A^{T}P\right)-cL^{2}⊗2PBB^{T}P\right]\bar{x}\\ \; & +\sum_{i=1}^{N}2\eta_{i}^{T}PB\epsilon_{i}\left(t\right)-\sum_{i=1}^{N}2d\frac{\eta_{i}^{T}PBB^{T}P\eta_{i}}{∥K\eta_{i}∥+\varepsilon_{i}\left(t\right)}-\sum_{i=1}^{N}2\Pi_{i}\varepsilon_{i}. \end{array}$$

By Assumption 1, one has

$$\begin{array}{c} 2\eta_{i}^{T}PB\epsilon_{i}\left(t\right)\leq 2\Pi_{i}∥B^{T}P\eta_{i}∥. \end{array}$$

Thus,

(19) $$\begin{array}{cc} {\dot{V}}_{1}\leq & {\bar{x}}^{T}\left[L⊗\left(PA+A^{T}P\right)-cL^{2}⊗2PBB^{T}P\right]\bar{x}\\ \; & -2\sum_{i=1}^{N}\left[\frac{d\eta_{i}^{T}PBB^{T}P\eta_{i}}{∥B^{T}P\eta_{i}∥+\varepsilon_{i}\left(t\right)}+\Pi_{i}\varepsilon_{i}-\Pi_{i}∥B^{T}P\eta_{i}∥\right]\\ = & {\bar{x}}^{T}\left[L⊗\left(PA+A^{T}P\right)-cL^{2}⊗2PBB^{T}P\right]\bar{x}\\ \; & -2\sum_{i=1}^{N}\frac{\left(d-\Pi_{i}\right)\eta_{i}^{T}PBB^{T}P\eta_{i}+\Pi_{i}\varepsilon_{i}^{2}\left(t\right)}{∥B^{T}P\eta_{i}∥+\varepsilon_{i}\left(t\right)}. \end{array}$$

By noting $c\geq \frac{1}{\lambda_{2}\left(L\right)}$ and $d\geq \Pi_{i},\forall i\in V$, one can derive

(20) $$\begin{array}{cc} {\dot{V}}_{1}\leq & {\bar{x}}^{T}\left[L⊗\left(PA+A^{T}P-2PBB^{T}P\right)\right]\bar{x}\leq 0, \end{array}$$

where the last inequality is obtained from the LMI (12).

Therefore, one can know from (20) that $V_{1}\left(t\right)$ is bounded, and so are η and ${\tilde{W}}_{i}$. Following the well-known Barbalat’s Lemma [21], it is not difficult to derive that the formation error $\eta_{i}$ converges to zero, i.e., the formation is achieved under the controller (11). □

**Remark** **4.** *To realize the asymptotic convergence of the formation error for networked systems in the presence of unknown nonlinearities, the neural network approach is introduced, with the adaptive gain ${\hat{W}}_{i}$ designed to estimate the unknown neural network constant weight matrix. Moreover, an extra term is introduced in the controller to tackle the residual error.*

**Remark** **5.** *Compared with existing formation results [4,5,6,7,8,10,11], the proposed output-feedback formation controller depends on only relative output information, where no observer information transmission is needed during the whole process. Such a design structure has the advantages of reducing communication cost and being free from network attack.*

**Remark** **6.** *Note that the Lyapunov function $V_{1}$ involves the Laplacian matrix of the graph, indicating that the proposed distributed formation controller is applicable to only undirected graphs. For the case of the directed graph, it is much more difficult to present the distributed formation controller for the networked systems with unknown nonlinearities, due to the asymmetric property of the Laplacian matrix associated with the directed graph.*

**Remark** **7.** *Note that the proposed distributed formation controller requires the accurate relative output measurement. For the case in which measure errors exist, the estimation error of the formation error cannot accurately converge to zero at appointed time. However, it is not difficult to derive that the estimation error of the formation error is bounded if the measure error for each agent is bounded. Then, the proposed distributed formation controller can ensure the boundedness of the formation error for networked systems in the presence of measurement errors.*

## 4. Simulation

**Figure 1:**
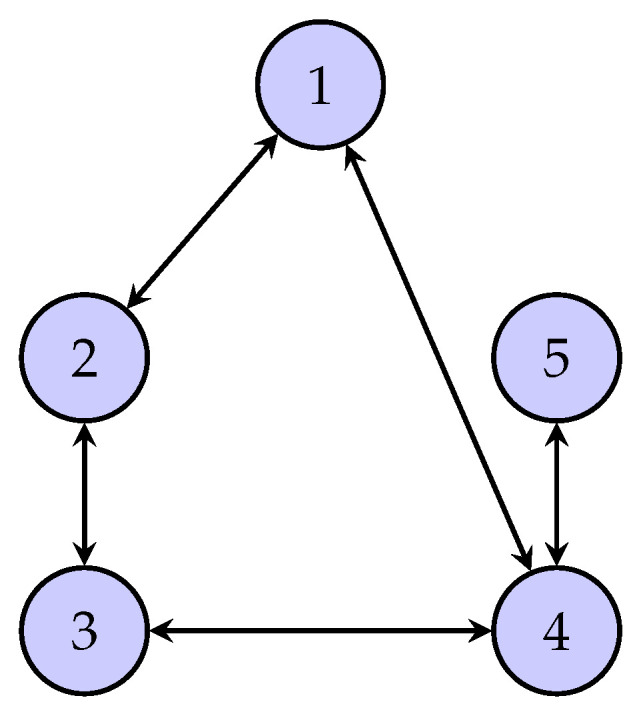
The communication topology among the five agents.

In this section, a numerical simulation is presented to demonstrate the effectiveness of the proposed controller. Consider the networked system consisting of five agents, the Laplacian topology among which is depicted in Figure 1. System matrices are given by (1) with

$$A=\left[\begin{array}{cccc} 0 & 1 & 0 & 1\\ 0 & -1 & 1 & 0\\ 1 & 0 & 0 & 1\\ -1 & 0 & 2 & 1 \end{array}\right],\;B=\left[\begin{array}{cc} 0 & 0\\ 1 & 0\\ 0 & 0\\ 0 & 1 \end{array}\right],$$

$$C=\left[\begin{array}{cccc} 0 & 0 & 1 & 0\\ 0 & 0 & 0 & 1\\ 1 & 1 & 0 & 1 \end{array}\right],$$

which satisfy Assumption 2. The unknown nonlinearities are presented as $f_{i}\left(t\right)=\left[\begin{array}{c} 0\\ f_{i2}\left(t\right) \end{array}\right]$ with $f_{i2}=\sin\left(\frac{t-1}{t+1}\right)-i\cos\left(\frac{t}{t+2}\right)-2\left(i+1\right)\sin\left(\frac{2(t-1)}{t+1}\right)+2\cos\left(\frac{2t}{t+2}\right)+\cos\left(2i\right)+e^{1-t}$, where the basic functions $\varphi_{i}\left(t\right)={\left[\sin\left(\frac{t-1}{t+1}\right),\cos\left(\frac{t}{t+2}\right),\sin\left(\frac{2(t-1)}{t+1}\right),\cos\left(\frac{2t}{t+2}\right)\right]}^{T}$; the unknown constant matrix $W_{i}=\left[\begin{array}{cccc} 0 & 0 & 0 & 0\\ 1 & -i & -2(i+1) & 2 \end{array}\right]$; and the residual error $\epsilon_{i}\left(x_{i},t\right)=\left[\begin{array}{c} 0\\ \cos\left(2i\right)+e^{1-t} \end{array}\right]$ with upper bound $\Pi_{i}=1+e$.

Choose

$$B_{0}=\left[\begin{array}{cc} 1 & 0\\ 0 & 0\\ 0 & 1\\ 0 & 0 \end{array}\right],\;C_{0}=\left[\begin{array}{c} 1\\ 0\\ 0 \end{array}\right].$$

Then, one can obtain

$$T=\left[\begin{array}{cccc} 1 & 0 & 0 & 0\\ 0 & 0 & 1 & 0 \end{array}\right],\;S=\left[\begin{array}{cccc} 0 & 1 & 0 & 0\\ 0 & 0 & 0 & 1 \end{array}\right],$$

and

$$V=\left[\begin{array}{ccc} 1 & 0 & 0 \end{array}\right],\;U=\left[\begin{array}{ccc} 0 & -1 & 1\\ 0 & 1 & 0 \end{array}\right].$$

Further, choose $F=\left[\begin{array}{c} -4\\ 3 \end{array}\right]$, which gives

$$\bar{A}=\left[\begin{array}{cc} 1 & 4\\ -1 & -3 \end{array}\right].$$

Solving the LMI (12) gives

$$Q=\left[\begin{array}{cccc} 1.0750 & 0.0078 & 0.2377 & -0.5300\\ 0.0078 & 0.5077 & 0.1902 & -0.1939\\ 0.2377 & 0.1902 & 0.4540 & -0.6289\\ -0.5300 & -0.1939 & -0.6289 & 1.0284 \end{array}\right],$$

and

$$P=\left[\begin{array}{cccc} 1.4408 & 0.0104 & 1.7846 & 1.8360\\ 0.0104 & 2.5179 & -2.5876 & -1.1023\\ 1.7846 & -2.5876 & 19.3099 & 12.2407\\ 1.8360 & -1.1023 & 12.2407 & 9.1966 \end{array}\right],$$

which yields

$$K=\left[\begin{array}{cccc} -0.0104 & -2.5179 & 2.5876 & 1.1023\\ -1.8360 & 1.1023 & -12.2407 & -9.1966 \end{array}\right].$$

The formation configuration is set as

$$p_{14}=\left[\begin{array}{c} 3\\ 3\\ -1\\ -3 \end{array}\right],\;p_{21}=\left[\begin{array}{c} 1\\ 1\\ 1\\ -1 \end{array}\right],\;p_{32}=\left[\begin{array}{c} -3\\ -3\\ 1\\ 3 \end{array}\right],$$

$$p_{43}=\left[\begin{array}{c} -1\\ -1\\ -1\\ 1 \end{array}\right],\;p_{54}=\left[\begin{array}{c} 2\\ 2\\ 0\\ -2 \end{array}\right],$$

which satisfies condition (2). Choose ${\bar{u}}_{i}$ as

$${\bar{u}}_{1}=\left[\begin{array}{c} -2\\ -4 \end{array}\right],\;{\bar{u}}_{2}=\left[\begin{array}{c} -2\\ -4 \end{array}\right],\;{\bar{u}}_{3}=\left[\begin{array}{c} 2\\ 4 \end{array}\right],\;{\bar{u}}_{4}=\left[\begin{array}{c} 2\\ 4 \end{array}\right],\;{\bar{u}}_{5}=\left[\begin{array}{c} 0\\ 0 \end{array}\right].$$

**Figure 2:**
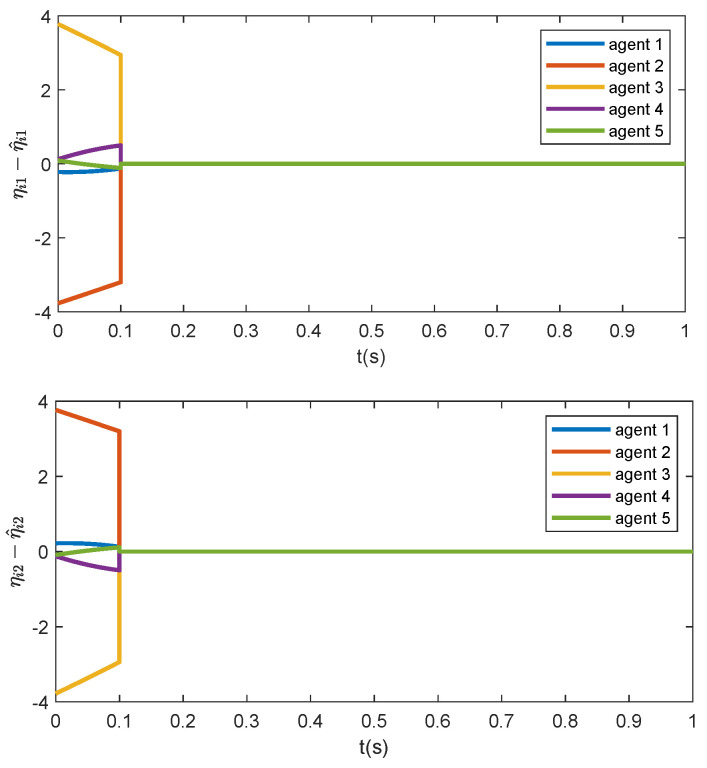

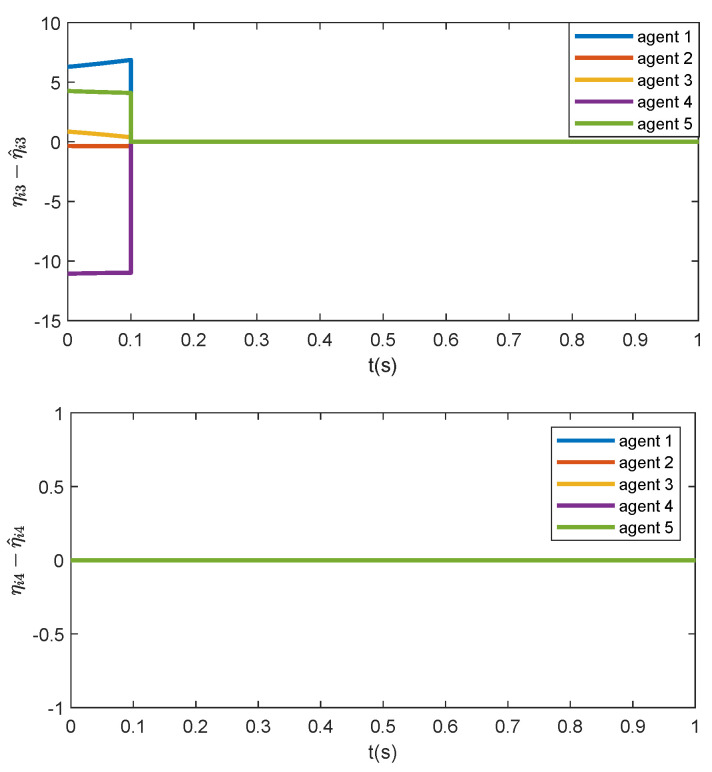
The trajectories of the estimation error $\eta_{i}\left(t\right)-{\hat{\eta }}_{i}\left(t\right)$.

**Figure 3:**
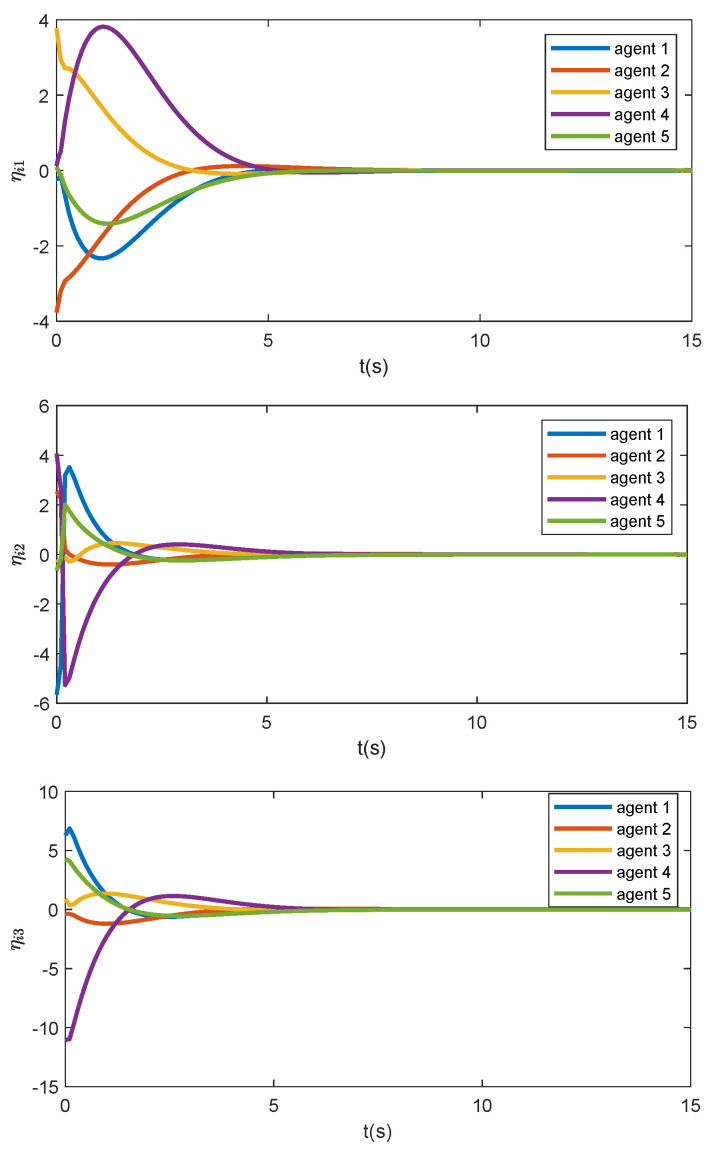

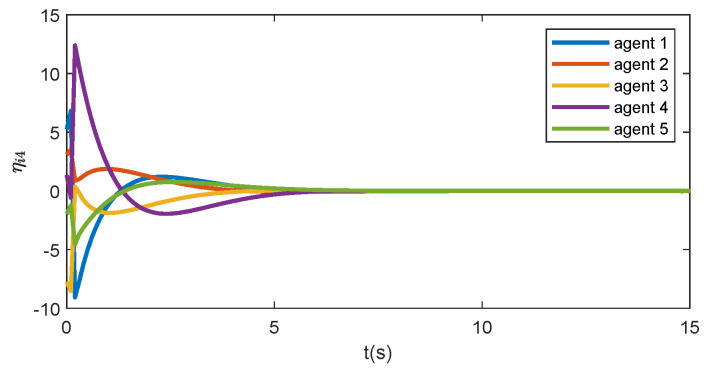
The trajectories of the formation error $\eta_{i}\left(t\right)$.

Furthermore, choose c=2, d=5 and $k_{i}=0.1$. To facilitate the simulation illustration, set the predefined time as $T_{0}=0.1$, and let u=0 before the predefined time $T_{0}$. Moreover, to avoid the singularity caused by G(0)=0, let ${\hat{\zeta }}_{i}\left(t\right)\equiv 0,\forall t\in \left[0,\tau \right)$. The initial states are set as [0,5]×[0,5]×[0,5]×[0,5] randomly. The trajectories of the estimation error $\eta_{i}\left(t\right)-{\hat{\eta }}_{i}\left(t\right)$ are presented in Figure 2. To show the effectiveness of the proposed appointed-time observer, only the trajectories of the estimation errors within time range [0,1] are drawn. Clearly, the proposed observer can estimate the formation error at predefined time τ=0.1. Note that

$$\left(I_{4}-BUC\right)B_{0}T=\left[\begin{array}{cccc} 1 & 0 & 0 & 0\\ -1 & 0 & 0 & 0\\ 0 & 0 & 1 & 0\\ 0 & 0 & 0 & 0 \end{array}\right],$$

indicating that $\eta_{i4}\left(t\right)-{\hat{\eta }}_{i4}\left(t\right)\equiv 0$. This is consistent with the simulation result as shown in Figure 2. The trajectories of the formation error are illustrated in Figure 3, which asymptotically converge to zero, meaning that the formation of the five agents can be achieved under the proposed distributed formation controller.

## 5. Conclusions

In this paper, the NN-based distributed formation controller was proposed based on the reduced-order observer. The designed reduced-order observer can estimate the formation error of networked systems based on only relative output information at any predefined time, and the order reduction in the observer is mainly realized by the transformation *T*. In the future, the following directions can be further investigated.

- The distributed formation controller presented in this paper is applicable to undirected connected graphs. In practice, the graphs may be general directed, and thus, it is preferable to design distributed formation controllers of networked systems under directed graphs based on the reduced-order appointed-time observer.
- The distributed formation controller presented in this paper continuously changes. In the real world, it is more desirable to design discrete-time controllers, which leads to the investigation of the event-triggered formation controller for networked systems based on the reduced-order appointed observer.
- The distributed formation controller presented in this paper depends on the agents’ dynamic model. In practice, the nominal model is difficult to obtain, and it is welcome to design data-driven appointed-time observers and formation controllers without using the agents’ dynamic model.
- The distributed formation controller presented in this paper can ensure the asymptotical convergence of the formation error for networked systems in the absence of disturbances. It is preferable to analyze the robustness of the appointed-time observer-based formation controller for networked systems in the presence of disturbances by theoretically revealing the upper bound of the formation error.
- The parameters of the distributed formation controller depend on the connectivity of the graph, which is not fully distributed. It is desirable to design a fully distributed formation controller based on appointed-time observers by introducing adaptive gain to estimate the global information of the graphs.
- The formation shape in this paper is fixed. One can further study the distributed appointed-time observer-based formation controller for time-varying formation tasks.

## Data Availability

Data are contained within the article.

## Abbreviation

The following abbreviation is used in this manuscript:

NN	neural network
